# Quercetin Protects Blood–Brain Barrier Integrity and Maintains Microvascular Permeability Following Traumatic Brain Injury

**DOI:** 10.1007/s12028-025-02315-z

**Published:** 2025-07-30

**Authors:** Bobby D. Robinson, Antonia Yeager, Angela Lomas, Gabriela Seplovich, Chinchusha Anasooya Shaji, Katie Wiggins-Dohlvik, Jason H. Huang, Claire L. Isbell, Binu Tharakan

**Affiliations:** 1 Department of Surgery, Baylor Scott & White Health & Texas A&M University College of Medicine, Temple, TX USA; 2 Texas Bioscience Institute, Temple, TX USA; 3 Department of Neurosurgery, Baylor Scott & White Health & Texas A&M University College of Medicine, Temple, TX USA; 4 Department of Surgery, Morehouse School of Medicine, Atlanta, GA 30310 USA

**Keywords:** Quercetin, Blood–brain barrier dysfunction, Traumatic brain injury, Microvascular hyperpermeability, Tight junction proteins, Antioxidant, Endogenous antioxidants, Zonula occludens-1, VE-cadherin, Immunofluorescence, *F*-actin, Brain microvascular endothelial cells, Reactive oxygen species

## Abstract

**Background:**

Cerebral edema is a consequential outcome of traumatic brain injury (TBI) and may lead to intracranial hypertension, necessitating urgent medical attention. One of the primary causes of cerebral edema is microvascular hyperpermeability, characterized by excessive leakage of intravascular fluid and proteins via blood–brain barrier (BBB) dysregulation. Prolonged activation of reactive oxygen species (ROS) formation and inflammatory pathways due to BBB hyperpermeability results in poor patient outcomes. The primary goal of this study was to ascertain if quercetin, a bioflavonoid plant pigment, would protect against BBB breakdown and hyperpermeability in the acute context following TBI.

**Methods:**

We used a mixed in vitro and in vivo model to test the effects of quercetin pretreatment on endothelial cell tight junctions in murine models of TBI and stress-induced hyperpermeability. Hydrogen peroxide (H_2_O_2_), a key contributor of secondary injuries following TBI, was used as an inducer of oxidative stress in cerebral endothelial cells in vitro. BBB tight junction/cytoskeletal integrity was assessed using immunofluorescence of junctional proteins zonula occludens-1, β-catenin, and vascular endothelial–cadherin, alongside filamentous actin labeling and a monolayer permeability assay. Intracellular ROS and H_2_O_2_ levels were determined using fluorescent probes. In vivo experiments consisted of intravital microscopy of brain pial vasculature in a mouse model of TBI.

**Results:**

The results demonstrate that quercetin (100 μM; 1 h) attenuated H_2_O_2_ (100 μM; 2 h)–induced monolayer hyperpermeability and ROS formation significantly and decreased the loss of tight junction and cytoskeletal integrity. Quercetin treatment (50 mg/kg) after injury decreased TBI-induced vascular hyperpermeability significantly compared to sham. These results indicate that quercetin provides BBB protection by decreasing oxidative stress–induced loss of tight junction/cytoskeletal integrity, ultimately resulting in decreased microvascular hyperpermeability.

**Conclusions:**

The data suggest that quercetin may be a viable therapeutic option for preventing or managing cerebral oedema acutely following TBI.

## Introduction

Traumatic brain injury (TBI) is a leading cause of disability and death worldwide. Sixty-nine million individuals worldwide are estimated to sustain a TBI [1], with increased incidence in both very young and older adults [2–6], athletes, [7] veterans [8, 9], and survivors of domestic violence [1]. Cerebral edema, the pathological accumulation of excess fluid in the central nervous system, is an urgent consequence following TBI leading to an elevation of intracranial pressure that can contribute to brain death if not treated promptly and appropriately [10–13].

The primary injury of TBI is due to a direct and immediate mechanical disruption of brain tissue and the surrounding microvascular [14]. Secondary injury gives rise to multiple parallel and interacting signaling pathways that mediate changes in neuronal, glial, and vascular structures [2, 10, 14, 15], the former of which is the focus of this study. Damage to cerebral microvasculature is known to induce oxidative stress by the formation of excessive reactive oxygen species (ROS), including hydrogen peroxide (H_2_O_2_) [16, 17], as well as the activation of proteolytic enzymes such as matrix metalloproteinase-9 (MMP-9), which induce blood–brain barrier (BBB) disruption [11, 13, 14, 18, 19]. Recent studies from our laboratory and by others have shown that TBI results in the endogenous formation and release of H_2_O_2_, with deleterious impacts on BBB integrity [8, 10, 19–21].

The BBB is a semipermeable membrane that separates the systemic circulation from the brain parenchyma. Its optimal functioning is crucial for the maintenance of brain homeostasis and overall protection of the brain from harmful substances [8]. The innermost component of the BBB is a protective layer of endothelial cells that are held together by tight junction proteins, including zonula occludens-1 (ZO-1) and claudins and adherens junction proteins, β-catenin, and vascular endothelial–cadherin (VE-cadherin). These endothelial cells are reinforced by surrounding pericytes and astrocyte processes, and an inner and outer acellular basement membrane. TBI-induced BBB compromise manifest initially as increased barrier permeability with delocalization and reduced expression of tight junction proteins that may progress to pericyte detachment, astrocyte end-feet swelling or loss, and a disrupted basement membrane. Disruption of the BBB eventually culminates in cerebral edema and elevated intracranial pressure leading to serious clinical complications such as brain herniation, permanent neurological dysfunction, and cerebral death [10, 14, 22]. Currently, there are no Food and Drug Administration (FDA) pharmacological therapies that have been approved for acute treatment of TBI. Uncovering how to mitigate TBI-associated cerebral edema is critical to the development of therapeutics and to improve patient outcomes. 

Quercetin (2-[3,4-dioxidophenyl]-3,4-dioxo-3,4-dihydro-2H-1-benzopyran-5,7-bis[olate]) is a polyphenolic bioflavonoid found commonly in herbs, fruits, and vegetables. It is a plant pigment and a phytoestrogen. Quercetin has many proposed functions including free radical scavenging and induction of antioxidant defense via nuclear factor erythroid-derived-like 2 (Nrf2) activation; anti-inflammatory properties via nuclear factor kappa-β (NF-κβ) inactivation; downregulation of MMP-9 [23]; and antiapoptotic properties through c-Jun N-terminal kinase modulation and extracellular-signal-regulated kinase pathways [24, 25]. Given these functions, quercetin has the potential to inhibit oxidative stress and proinflammatory pathways [26–28] and thus may enhance BBB function acutely following TBI.

The objectives of this study were to determine (1) whether quercetin would protect the BBB against TBI-induced BBB breakdown and hyperpermeability in vivo, (2) whether quercetin would protect the BBB against oxidative stress–induced BBB breakdown and hyperpermeability in vitro, and (3) whether the protective effects of quercetin are mediated via protection of BBB tight junction associated proteins and actin cytoskeleton.

## Methods

### Materials

Quercetin ≥ 95% (high-performance liquid chromatography) was purchased from Sigma-Aldrich (St. Louis, MO) and dissolved in dimethyl sulfoxide (DMSO) 99.5% purchased from ThermoFisher (Carlsbad, CA). The concentration of DMSO was kept to less than 0.1% to avoid cell toxicity. Rat brain microvascular endothelial cells (RBMECs) and RBMEC medium were obtained from Cell Applications Inc. (San Diego, CA). Transwell plates were obtained from Corning Costar (Corning, NY). Nunc Lab Tek II-CC, 8-well glass chamber slides, fibronectin from bovine plasma, albumin from bovine serum, and fluorescein isothiocyanate fluorescein isothiocyanate (FITC)-dextran-10 kDa were purchased from Sigma-Aldrich. Rabbit anti ZO-1 (catalog no. 617300), 0.25% Trypsin, Opti-MEM reduced serum medium, Dulbecco’s modified Eagle’s medium (DMEM) (with high glucose, 4-[2-hydroxyethyl]-1-piperazineethanesulfonic acid, no phenol red and rhodamine phalloidin were purchased from Thermo Fisher Scientific (Carlsbad, CA). VECTASHIELD Mounting Media with 4′,6-diamidino-2-phenylindole (DAPI) was purchased from Vector Laboratories (Burlingame, CA). A Hydrogen Peroxide Intracellular Assay Kit and a Intracellular Reactive Oxygen Species Kit were bought from BioVision (Milpitas, CA). VE-cadherin C-19 antibody (sc-6458) and β-catenin H102 (sc-7199) primary antibodies were purchased from Santa Cruz Biotechnologies (Santa Cruz, CA). 

### Cell Cultures

Primary cultures of RBMECs derived from the brain of adult Sprague-Dawley rats were purchased from Cell Applications Inc. RBMECs were initially grown on 0.05% fibronectin-coated cell culture dishes using the RBMEC medium in a cell culture incubator (95% O_2_ and 5% CO_2_ at 37 °C). For cell detachment, endothelial cells were treated with 0.25% trypsin– ethylenediaminetetraacetic acid. Detached cells were then grown on fibronectin-coated Transwell inserts, chamber slides, or 100-mm dishes for experimental purposes. RBMEC passages greater than 12 were not used for the experiments. 

### Immunofluorescence Localization of Tight Junction Associated Proteins

The purpose of using immunofluorescence (IF) was to understand if there was displacement or delocalization of junctional proteins between cerebral endothelial cells in a simple, simulated BBB monolayer. ZO-1, β-catenin, and VE-cadherin junctional localization were assessed. Filamentous actin (*F-*actin) stress fibers were visualized to investigate if there were changes in intracellular cytoskeletal structural integrity. Rhodamine phalloidin labeling technique was used for visualization. RBMECs were grown on chamber slides overnight. Cells were initially exposed to Opti-MEM/reduced serum medium. The experimental groups included an untreated control, H_2_O_2_ (100 µM for 2 h), quercetin (100 µM for 1 h + H_2_O_2_; 100 µM for 2 h), and quercetin (100 µM for 1 h). Our previous studies show that H_2_O_2_ at 100 µM for 2 h induces endothelial cell monolayer hyperpermeability while maintaining cell viability [17]. Thus, H_2_O_2_ is used a positive control, inducing barrier dysfunction without losing cell viability [17]. The concentration and exposure duration of quercetin was chosen after a review of the literature focusing on in vitro studies investigating the effects of quercetin on cell culture models of disease [24–31]. Cells were then fixed in 4% paraformaldehyde in phosphate-buffered saline (PBS) for 10–15 min and permeabilized in 0.5% Triton-X 100 in PBS for another 10–15 min. Cells were blocked using 2% bovine serum albumin (BSA) in PBS for 1 hour at room temperature. Cells were incubated overnight in anti-rabbit primary antibodies against ZO-1 (1:150) in 2% BSA-PBS, followed by incubation with anti-rabbit IgG-FITC conjugated secondary antibody for 1 hour at room temperature. Cells were washed and mounted using VECTASHEILD Antifade Mounting Media with DAPI for nuclear staining. This process was repeated with β-catenin (1:150) and VE-cadherin (1:150) primary antibodies followed by anti-rabbit and anti-goat IgG-FITC conjugated secondary antibodies, respectively. Cells were visualized and scanned at a single optical plane with an Olympus Fluoview 300 Confocal Microscope (Center Valley, PA), with a PLA PO 60X water immersion objective. The images were analyzed with ImageJ software (National Institutes of Health) to quantify mean junctional fluorescent intensity across groups. 

### ImageJ Analysis

ImageJ software [32] was used to quantify in vitro IF images and in vivo intravital microscopy. Images were converted to 8-bit grayscale and background subtraction was performed. Regions of interest were manually selected and corrected total cell fluorescence was determined using the following formula: corrected total cell fluorescence = integrated density—(area × mean background fluorescence). Results are expressed as relative fluorescence units (RFUs). All analyses were performed on three images per group with identical processing and settings applied throughout.

### Visualization of Cytoskeletal Organization

For the rhodamine phalloidin labeling of *F*-actin, treatments were performed as described previously using the same groups: untreated control, H_2_O_2_ (100 µM for 2 h), quercetin (100 µM for 2 h), and quercetin (100 µM for 1 h). Following treatment, cells were fixed, permeabilized, and blocked in 2% BSA-PBS. Cells were then labeled with rhodamine phalloidin (1:50) in 2% BSA-PBS for 20 min. Chamber slides containing cells were washed and mounted using VECTASHIELD antifade Mounting Media with DAPI for nuclear staining. Cells were visualized and scanned at a single optical plane with an Olympus Fluoview 300 Confocal Microscope, with a PLA PO 60X water immersion objective. Untreated cells served as controls; the H_2_O_2_ group served as a positive control, as described previously. After imaging with the confocal microscope, images were analyzed using ImageJ software to quantify mean actin fiber fluorescent intensity and compared using the statistical methods described in the following section.

### Monolayer Permeability Assay

Rat brain microvascular endothelial cells were grown on fibronectin-coated Transwell inserts as monolayers for 72–96 h and checked daily for confluency. Monolayers were initially exposed to phenol red free DMEM for 45 min to an hour. The experimental groups included an untreated control, H_2_O_2_ (100 µM for 2 h), quercetin (100 µM for 1 h) + H_2_O_2_ (100 µM for 2 h), and quercetin (100 µM for 1 h). At the end of the treatment, FITC labeled dextran of 10 kDa (5 mg/mL) was applied to the luminal compartment and leakage of this fluorescent compound was measured after an incubation period of 30 min at 37 °C to reflect permeability of the endothelial monolayer in experimental groups compared to control. A sample of 100 µL was collected from the abluminal compartment and measured fluorometrically at 485/520 nm (Excitation/Emission [Ex/Em]) using Fluoroskan Ascent FL Microplate Fluorometer and Luminometer (Vantaa, Finland). These methods have been previously described by our laboratory and others [17, 33, 34], wherein a relative increase in fluorescence suggests increased monolayer permeability. 

### Visualization of Intracellular H_2_O_2_ and ROS Formation

Rat brain microvascular endothelial cells were grown on a 96-well plate overnight as described previously. The treatment group was 100 µM quercetin for 1 h followed by 100 µM H_2_O_2_ for 2 h. The experimental groups included untreated control, vehicle control, H_2_O_2_ (100 µM for 2 h), quercetin (100 µM for 1 h) with H_2_O_2_ (100 µM for 2 h), and quercetin (100 µM for 1 h). The cells were then processed for the intracellular visualization of H_2_O_2_ using a BioVision Intracellular H_2_O_2_ Detection Kit and imaged using the Olympus Confocal Microscope (Ex/Em = 543 nm/545–750 nm). A BioVision Reactive Oxygen Species Detection Assay was performed to assess the quantity of ROS in each culture group. This technique detects intracellular levels of hydroxyl, peroxyl, or other ROS in live cells using a unique cell-permeable fluorogenic probe. Oxidation of this compound by intracellular ROS yields a highly fluorescent product that can be detected (Ex/Em 495/529 nm).

### Animals and Surgeries

Mice were chosen as the appropriate model to investigate TBI-induced oxidative stress pathophysiology on BBB structure and function. Male C57BL/6 mice (18–25 g) were purchased from Jackson Laboratory (Bar Harbor, ME). Male mice were used for homogeneity of population, and to be consistent with previous studies from our lab. Animals were maintained at the Texas A&M University Health Science Center College of Medicine and Baylor Scott and White Health animal facility on a 12:12 hour dark–light cycle, with free access to food and water but no food at midnight prior to surgery. The room temperature was maintained at 25 °C ± 2 °C. Surgical and experimental procedures used in this study were conducted after approval from the Institutional Animal Care and Use Committee. The facility is approved by the Association for Assessment and Accreditation of Laboratory Animal Care International in accordance with the National Institutes of Health guidelines. The animals were anesthetized with urethane, intraperitoneal (IP) injection (2 mL/kg body weight) and continuously observed by an investigator until the end of the study (up to 1 h following TBI). Our studies show that, at this dose of urethane, animals are under deep anesthesia and experience minimal suffering. This was not a survival study, and no unexpected animal deaths or adverse events were observed.

A midline incision on the scalp exposed the sagittal suture, bregma, and lambda. A circular craniectomy window, approximately 3–4 mm in diameter, was made over the right hemisphere, between lambda and bregma, using a microdrill. The resulting bone flap was removed. Sham animals received only craniectomy surgery, whereas TBI injury group received brain injury via Benchmark Stereotaxic Impactor from Leica (Leica Biosystems Inc., Buffalo Grove, IL). Following craniectomy procedure, the animals were mounted on the stereotaxic frame and an impactor probe of 3 mm diameter was used to impact the exposed part of the brain. The depth of the injury was used to determine the severity of the injury. Settings for moderate TBI used in this study were: 2-mm depth, 0.55-m/s velocity, and 100-ms contact time [35].

### Surgical Procedures and Intravital Microscopy

Animals undergoing intravital microscopy were divided into groups as follows: sham + vehicle, TBI + vehicle, and TBI + quercetin (*n *= 6). Power calculation is described in the following section. DMSO was used as the vehicle, and blood levels were kept at less than 0.1% to avoid toxicity. Animals undergoing TBI were anesthetized and underwent TBI as stated previously. The TBI + quercetin group received 50 mg/kg of quercetin dissolved in DMSO by tail vein injection 10 min after injury. Quercetin concentration was selected based off a review of the literature [29, 31, 36]. A no. 0 glass 5-mm round coverslip from Warner Instruments (Hamden, CT) was then adhered over the craniotomy site, and the mouse was placed on the platform mounted to the intravital fluorescent microscope (Nikon E600, Tokyo, Japan). The temperature was maintained at 37 °C with a heating pad. The animals received an intravenous bolus of FITC-dextran-10 kDa (0.1 mL of 50 mg/mL) prior to injury. Pial vessels of 50–75 µm were selected for analysis with a 40× objective. Images were captured at 10, 30, 50, and 70 min after TBI to assess for changes in fluid permeability over time. Images were obtained with a Photometrics Evolve Camera (Photometrics, Tucson, AZ) and captured digitally and analyzed using Nikon NIS-Elements Software. Qualitative images were subsequently quantified as RFUs using ImageJ analysis, as previously described.

#### Ex Vivo Brain Tissue Analysis

Animals were treated as described previously: sham + vehicle, TBI + vehicle, and TBI + quercetin (*n*=4). DMSO was used as the vehicle and blood levels were kept at less than 0.1% to avoid toxicity. The TBI + quercetin group received 50 mg/kg of quercetin dissolved in DMSO by tail vein injection 10 min after injury. Randomization and anonymizing were not used. Animals were killed after 1 h, and the brain tissue was removed intact.

#### Statistical Analysis and Power Calculation

Data are expressed as the mean ± percentage of the standard error of the mean for monolayer permeability, ROS assay, confocal microscopy data, and intravital microscopy data. Statistics were performed using GraphPad Prism 7 statistical software. A Student’s *t-*test was used to compare mean quantitative value between two groups. Analysis of variance was used to compare mean quantitative measures across more than two groups. Post hoc analysis was conducted using Bonferroni correction to adjust for multiple comparisons and reduce the probability of false positive errors. The open access software G*Power [37] was used to calculate the sample size needed to achieve a power of 80% and an alpha of 5% with three groups and four measurements per group. A large effect size of *d*=1.5 was predicted given the literature on moderate TBI in the acute time period [38, 39]. A sample size of 6 was generated using the following code:
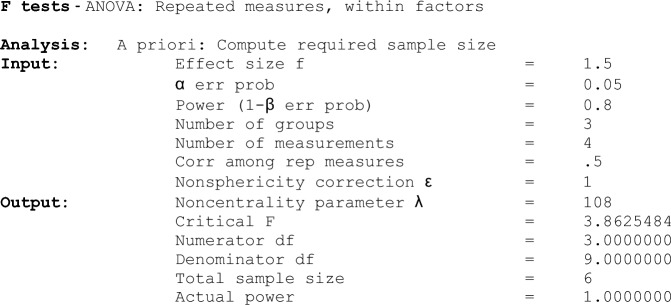


## Results

### Quercetin Protects the Tight Junction, Adherens Junction, and Cytoskeletal Integrity

**Fig. 1:**
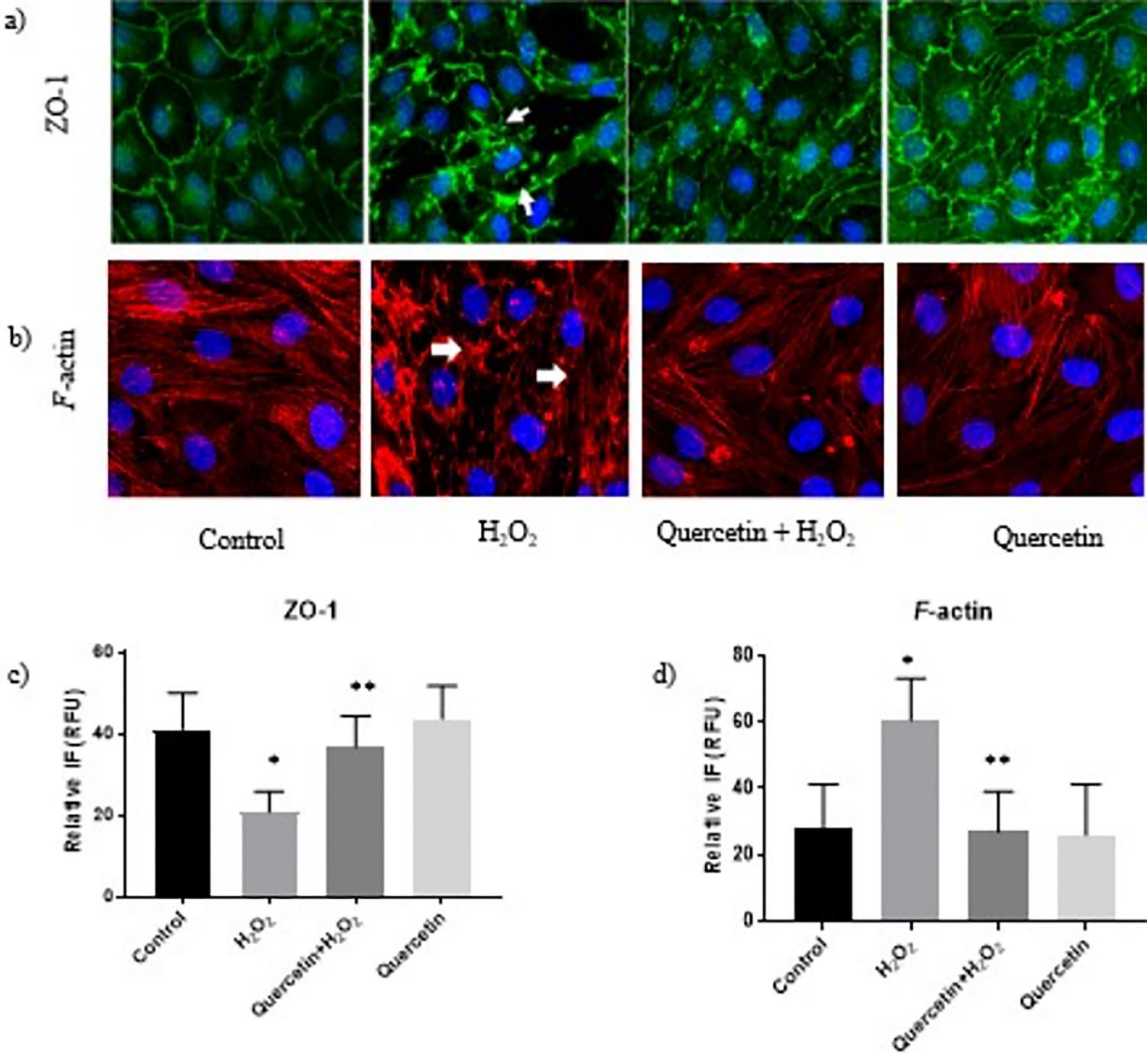
Immunofluorescence of Tight Junction Protein , ZO-1, and labeling of Actin Fibers in Rat Brain Microvascular Endothelial Cells. **a** Immunofluorescence localization of tight junction protein Zonula Occludens-1 (ZO-1). Arrows in pictures indicate tight junction disruption. **b** Rhodamine phalloidin labeling of *F*-actin demonstrating changes in the actin cytoskeleton. Arrows indicate the changes in cytoskeletal morphology. **c**, **d** ZO-1 junctional fluorescence and *F*-actin intensity was measured using ImageJ software and quantified and compared among groups. ‘*’ indicates statistically significant compared to control. ‘**’ indicates statistically significant compared to H_2_O_2_ group (*P *< 0.05)

Immunofluorescence localization of the tight junction associated junctional proteins provides qualitative data regarding BBB structural integrity at the cell-cell junctions. The purpose of this experiment was to investigate changes in junctional integrity across experimental groups in which untreated cells served as a negative control and H_2_O_2_ treatment alone served as a positive control. Figure 1a shows IF localization of ZO-1 in RBMECs (green staining). In control cells, ZO-1, β-catenin, and VE-cadherin showed continuous junctional localization, as expected, indicating healthy, adherent cells representing the innermost layer of the BBB. Conversely, the H_2_O_2_ treatment (100 µM; 2 h) group showed loss of continuous junctional localization, indicating loss of cell-to-cell junctional integrity. Quercetin treated (100 µM; 1 h) cells showed restoration of these tight junction proteins (Fig. 1a) suggesting protection from H_2_O_2_ induced disruption. ImageJ analysis, which converts image data into measurable RFUs to compare mean values across groups, indicates a statistically significant difference among treatment groups (Fig. 1c).

*F-*actin is an integral component of the cytoskeleton and is typically regularly patterned in normal cells, which can be seen in Fig. 1b (red staining) as the control group. Application of H_2_O_2_ (100 µM; 2 h) led to the loss of this cytoskeletal architecture with arrows indicating the formation of *F-*actin stress fibers, indicating induction of cellular stress. A statistically significant increase in the mean RFUs was observed in cell cultures treated with H_2_O_2_ compared to control (Fig. 1d; *P *< 0.05). Quercetin treated (100 µM; 1 h) cells showed a statistically significant decreased cytoskeletal disorganization (Fig. 1d), indicating a restoration of cellular architecture.

**Fig. 2:**
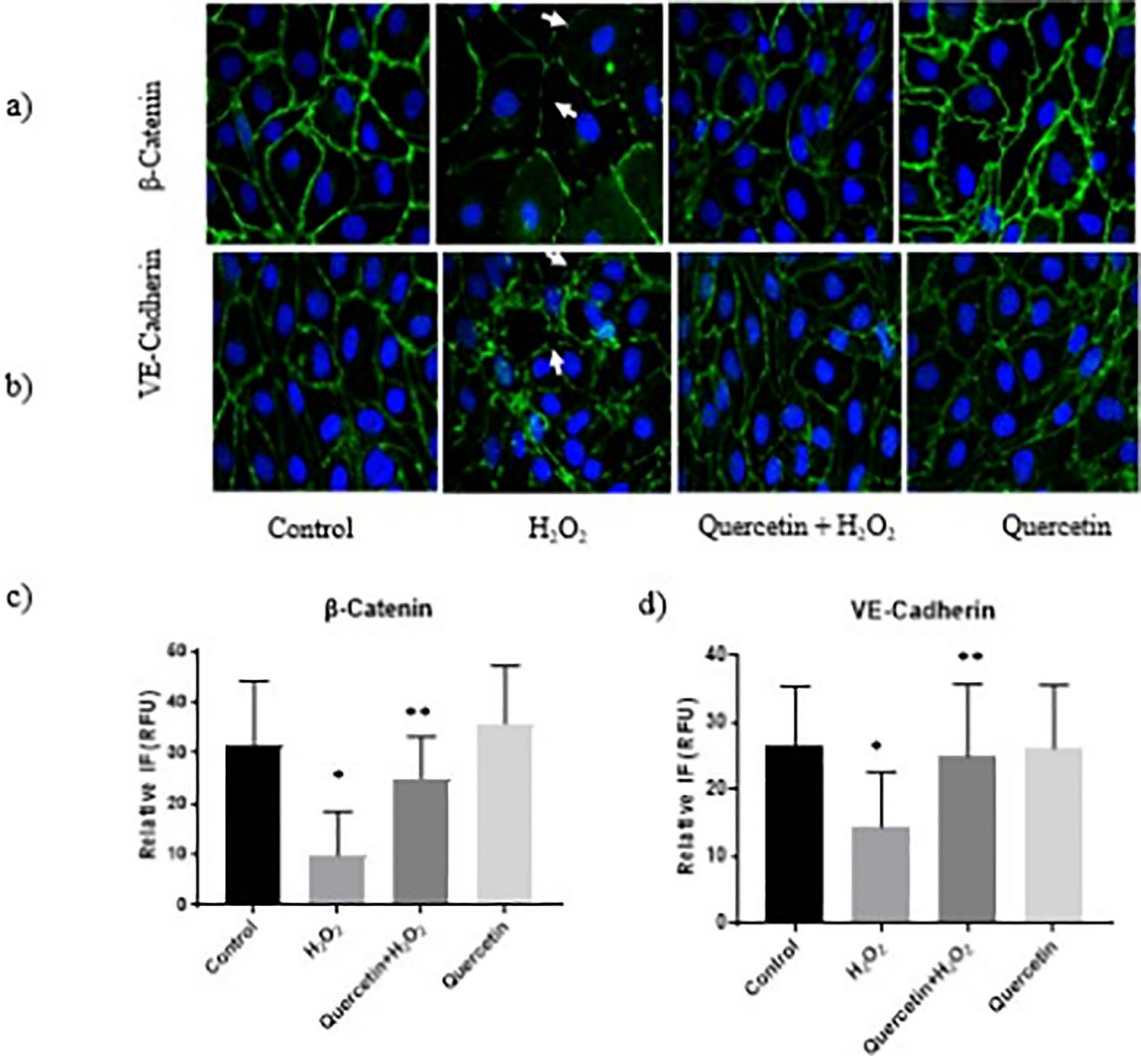
Immunofluorescence of Adherens Junction Proteins in Rat Brain Microvascular Endothelial Cells. **a** Immunofluorescence localization of adherens junction protein β-Catenin. **b** Immunofluorescence localization of adherens junction protein Vascular Endothelial Cadherin (VE-Cadherin). Arrows indicate adherens junction disruption. **c**, **d** β-Catenin and VE-cadherin junctional fluorescence intensity was measured using ImageJ software and quantified and compared among groups. ‘*’ indicates statistically significant compared to control. ‘**’ indicates statistically significant compared to H_2_O_2_ group (*P *< 0.05)

Similarly, Fig. 2a, b shows IF localization of two adherens proteins, β-catenin, and VE-cadherin. In control cells, β-catenin and VE-cadherin showed continuous junctional localization, indicating normal cellular architecture. The H_2_O_2_ treatment (100 µM; 2 h) led to a decrease at both adherens junctions, indicated by a loss of continuity across adjoining cells in both the β-catenin and VE-cadherin groups. Quercetin treated (100 µM; 1 h) cells showed restoration of these adherens junctional proteins, depicted by greater continuity of IF staining along cell membranes, as is seen in the control. When quantified via ImageJ, there was a statistically significant decrease in the RFUs of the adherens junctional fluorescence with H_2_O_2_ treatment groups compared with control, with restorations of these junctions when cells were pretreated with quercetin (Fig. 2c, d; *P *< 0.05).

### Quercetin Decreases Monolayer Hyperpermeability

**Fig. 3:**
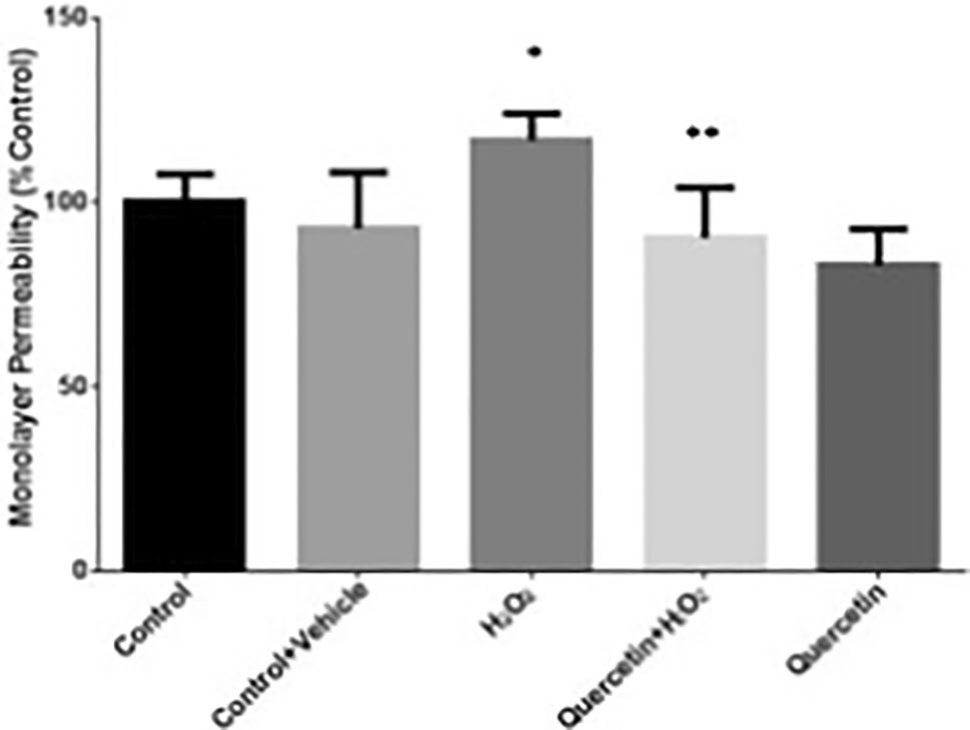
Monolayer Permeability Effects with H_2_O_2_ and Quercetin. Effect of quercetin on hydrogen peroxide (H_2_O_2_)-induced monolayer hyperpermeability in rat brain microvascualr endothelial cells. Quercetin significantly decreased H_2_O_2-_induced hyperpermeability compared to the H_2_O_2_ group. ‘*a*’ indicates statistically significant compared to control. ‘*b*’ indicates statistically significant compared to H_2_O_2_ group (*P *< 0.05)

Brain endothelial cells grown as monolayers in Transwell inserts create a functional endothelial barrier representing a minimalistic simulation of the innermost, protective layer of the BBB. H_2_O_2_ treatment (100 µM; 2 h) increased permeability compared to control, observed by an increase in FITC-dextran leakage from the apical to the basal compartment after a 30-minute incubation period (*P *< 0.05; Fig. 3). Prior treatment with quercetin (100 µM; 1 h) decreased H_2_O_2_-induced hyperpermeability significantly (*P *< 0.05; Fig. 3), represented by no significant difference in FITC-dextran leakage to control cells. Figure 3 shows the quantified results of FITC-dextran leakage, as compared with control, measured by a microplate fluorometer and luminometer.

### Quercetin Decreases Intracellular H_2_O_2_ and ROS Formation

**Fig. 4:**
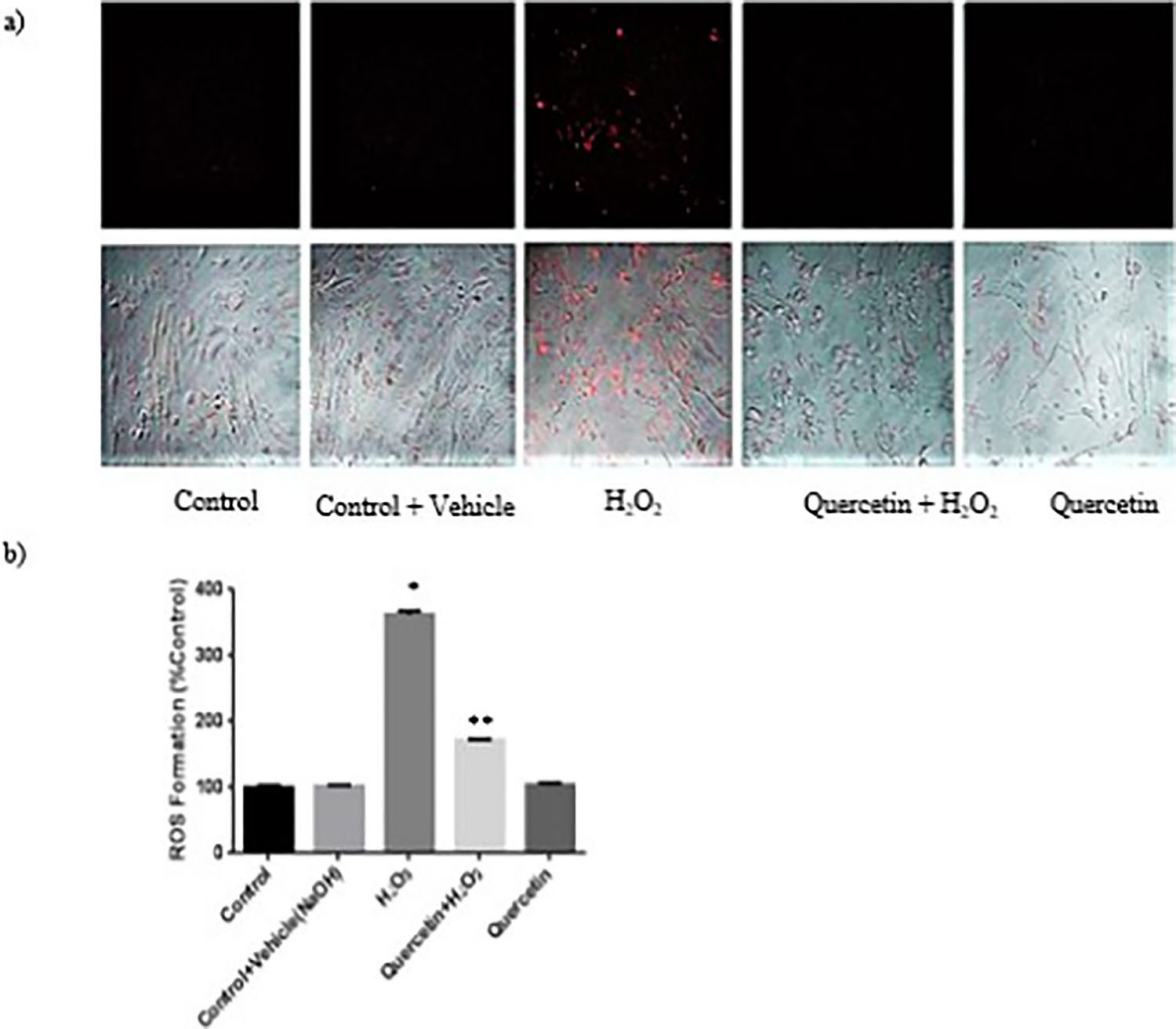
Effect of Quercetin on H_2_O_2_-Induced ROS Formation. **a** Qualitatively, there was in increase in H_2_O_2_ visualized with decrease with quercetin treatment. **b** An ROS formation assay was performed to quantify the effect of quercetin on ROS creation. Quercetin decreased the formation of ROS significantly. ‘*’ indicates statistically significant compared to control. ‘**’ indicates statistically significant compared to H_2_O_2_ group (*P *< 0.05)

One of the prominent features of quercetin as a therapeutic is its role as an antioxidant. The ROS assay showed that extracellular H_2_O_2_ treatment resulted in an increase in intracellular H_2_O_2_, which was quantitively reduced with quercetin treatment (Fig. 4a). H_2_O_2_ treatment (100 µM; 2 h) caused a statistically significant increase in ROS formation compared with untreated control cells (*P *< 0.05). Prior treatment of quercetin (100 µM; 1 h) in H_2_O_2_ treated cells decreased H_2_O_2_-induced ROS formation significantly (*P *< 0.05; Fig. 4b).

### Quercetin Decreases BBB Dysfunction and Microvascular Hyperpermeability Following TBI

**Fig. 5:**
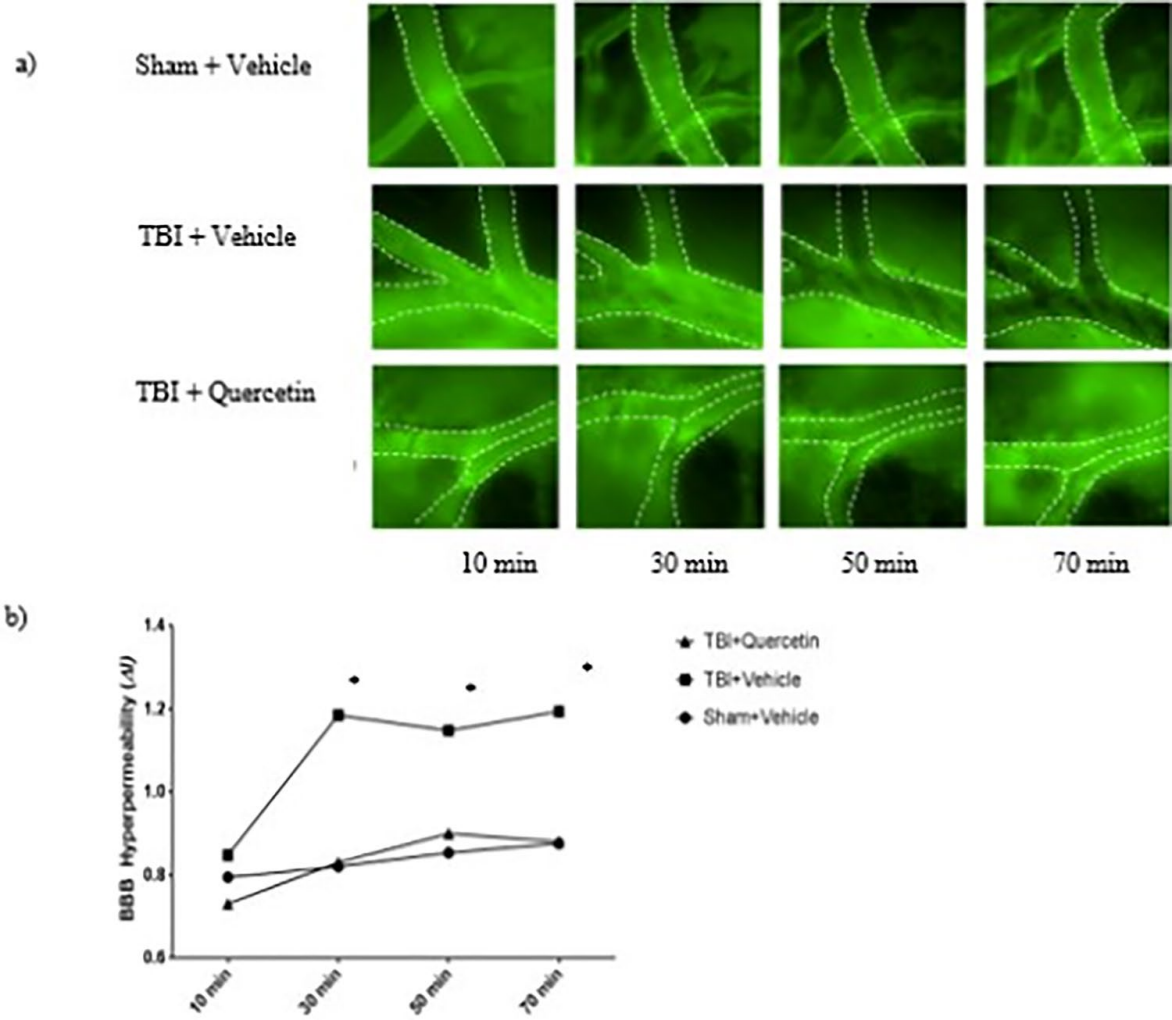
The Effects of TBI and Quercetin on Blood–Brain Barrier Function in a Mouse Model of TBI. **a** Representative images obtained from intravital microscopy of mouse brain pial vasculature in vivo. The venules analyzed have been outline for ease of visualization. **b** Graphical plotting of Δ*I,* a calculation of permeability. ‘*’ indicates statistical significance (*P *< 0.05). There was no difference among the groups at the initial time point (*P *= NS)

Intravital microscopy facilitates the real-time visualization of BBB dysfunction and microvascular permeability in live animals. Figure 5a shows representative images of pial microvasculature taken at 20-minute intervals (from minutes 0 to 70). These images show the progression of microvascular hyperpermeability in the TBI + vehicle group and attenuation of this effect in the TBI + quercetin group. There was a statistically significant decrease in microvascular hyperpermeability between the sham and TBI + quercetin groups and the TBI + vehicle group (*P *< 0.05), suggesting that quercetin treatment serves as a vascular protector, mitigating ROS-induced hyperpermeability. There was no statistically significant difference between groups at 10 min after injury (*P* = not significant). There was no significant difference between sham + vehicle and TBI + quercetin groups at 70 min (*P* = not significant).

## Discussion

### Summary of Findings

Traumatic brain injury is a major global public health threat that carries significant social and economic consequences. One of the major pathological mechanisms in TBI is the occurrence of vasogenic edema that leads to brain swelling, elevated intracranial pressure, and downstream neurological consequences, some of which may result in brain death. Despite decades of research, there is no FDA-approved treatment for TBI. The present study is relevant in that it demonstrates evidence for quercetin as a protectant of the BBB in the context of acute TBI. The major findings of this basic science study regarding quercetin are the following: (1) it is effective in decreasing ROS in cerebral endothelial cells; (2) it provides protection against the loss of BBB tight junction integrity by preserving tight junctions, adherens junction proteins, and actin cytoskeletal assembly in brain endothelial cells; (3) it maintains the critical barrier function of endothelial cells when exposed to oxidative stress; and (4) its intravenous administration has therapeutic potential against BBB breakdown and hyperpermeability in a mouse model of moderate TBI. These results indicate a potential application for quercetin as a clinical tool to diminish TBI-induced vasogenic oedema.

Prolonged oxidative stress due to formation of ROS is attributed as a major trigger for several adverse consequences of TBI, such as neuroinflammation and neurodegeneration. ROS, such as H_2_O_2_ and superoxide anions, should be quickly eliminated to prevent damage of cellular molecules and downstream neurological sequalae. Althoigj our findings are early and preclinical, they are clinically relevant in that they introduce the potential for a new compound to alleviate ROS-induced BBB hyperpermeability in acute TBI. Protecting and restoring the integrity of the BBB, particularly in the acute time point, is critical for preventing vasogenic oedema that can lead to brain swelling and intracranial hypertension. Ultimately, this study provides evidence of the potential for quercetin administration in the immediate aftermath following head trauma, which may protect against microvascular hyperpermeability.

### In Vitro Findings

Cerebral microvascular endothelial cells are the innermost protective barrier on the BBB. Their primary function is to provide a tight seal, minimizing exchange between the blood and the brain, thereby maintaining central nervous system homeostasis. These endothelial cells maintain a highly selective barrier, adhering to one another via tight junction and adherens junctional proteins, which include ZO-1, claudins, and occludens (tight junctions) and β-catenin and VE-cadheren (adherens junctions). Junctional integrity between endothelial cells is critical to the integrity of the BBB in both structure and function. Our cellular studies showed that quercetin protects against TBI-induced barrier disruption, minimizing tight junction and adherens junction dislocalization that alters endothelial functioning. We also showed that quercetin treatment helped maintain to restore the structural integrity of cerebral endothelial cells by reducing ROS-induced actin stress fiber formation on IF. Finally, through a barrier function assay, we showed that quercetin treatment can attenuate the microvascular hyperpermeability that normally follows exposure to ROS. Taken together, our cellular studies suggest that quercetin is effective in mitigating cellularly induced BBB disruption.

### In Vivo Findings

Our in vitro findings were supported by our in vivo findings*,* which included a controlled cortical impact model of moderate TBI. Cerebral edema, caused by vasogenic disruption of the BBB, can lead to potentially fatal intracranial hypertension. Both conditions represent a medical emergency in the context of acute head trauma. Our animal studies showed that ROS formation and oxidative stress, induced by a CCI model of TBI, can be significantly reduced when quercetin treatment is delivered within 10 min of injury. We also showed that quercetin significantly mitigates the pial vessel hyperpermeability normally induced by TBI. These findings provide further support for the identification of quercetin as a BBB protectant for acute TBI. 

### Possible Mechanisms for Quercetin Protection in Acute TBI

Quercetin, a flavanol compound, is known to improve the antioxidizing capacity of the human body [40] by increasing glutathione levels and thereby eliminating free radicals caused by cellular stress [40, 41]. Quercetin is also purported to inhibit proliferation, induce homeostatic apoptosis, and inhibit angiogenesis [42] and is being investigated for use in treating cardiovascular disease, pulmonary disease, inflammatory diseases, and diabetes [42]. Although the exact mechanism by which quercetin exerted protective effects on the BBB in our studies is not known, we discuss three pathways by which quercetin’s restorative effects may have occurred. These include downregulation of MMP-9, Nrf2 transcriptional upregulation, and nitric oxide (NO) signaling inhibition.

It has been reported that quercetin administration decreases levels of MMP-9, an enzyme within the family of metalloproteinases [43]. MMP-9 is widely studied and known to degrade the extracellular matrix and activate chemokines and cytokines that regulate tissue remodeling [44]. Several studies indicate a role for MMP-9 in neurological decline and BBB hyperpermeability, disrupting endothelial junctional proteins that can trigger inflammatory and degenerative processes. MMP-9 is initially secreted as pro-MMP-9, and thus requires activation. More than one pathway may activate MMP-9 cellular remodeling, including tumor necrosis factor alpha (TNF-α) and NF- κB [45], both of which are known to be activated following TBI [14]. Although proinflammatory processes are necessary for TBI recovery, prolonged activation of TNF-α and NF-κβ may lead to pathologically activating MMP-9 and prolonging junctional disruption. Other studies have demonstrated that quercetin may block TNF-α and NF-κβ [46], lessening damage at tight junctions and adherens junctions by limiting MMP-9 activity. 

Separately, quercetin is a known activator of cellular defense mechanisms via Nrf2 transcriptional upregulation, which, when activated, participates in detoxification and reduces ROS species [41]. In vivo studies have shown that Nrf2 knockout mice have worse functional outcomes following TBI, suggesting a key role for Nrf2 in head trauma. Clinical studies support these findings, wherein Nrf2 activation, in conjunction with Nicotinamide adenine dinucleotide phosphate oxidase 2 (NOX2) inhibition, result in improved motor and cognitive functioning, as well as decreased cortical contusion volume, in human patients with TBI. Therefore, by upregulating Nrf2, quercetin may prove to be clinically useful in helping eliminate toxic ROS, such as H_2_O_2_, to prevent the oxidative damage caused in cerebral endothelial cells following TBI. 

The NO signaling pathway is another mechanism by which quercetin may act as a cerebral endothelial cell protectant following TBI. NO signaling regulates cerebral blood flow under physiological and pathological conditions, including in the context of head trauma. NO regulation becomes dysregulated following TBI, leading to vascular consequences that include loss of autoregulation and neurovascular uncoupling, both of which may result in BBB hyperpermeability. Although the literature presents conflicting results, some studies show that quercetin attenuates NO production [47]. Thus, the NO signaling pathway may present another area of study for understanding the mechanism by which BBB functional outcomes are restored in our studies.

### Quercetin Pharmacokinetics

Quercetin is a flavonoid compound not produced endogenously in the human body. It is, however, readily present in the average diet because it is found in several fruits, vegetables, and herbs including apples, berries, broccoli, onions, pepper, capers, dill, and green tea. Because of several studies reporting anti-inflammatory effects, antioxidizing properties, and improved immune functioning [48], commercial availability of quercetin as a dietary supplement has increased. When consumed as part of the diet, quercetin is ingested as a glycoside and mostly converted into aglycone via glycosidic cleavage in the intestine before it can be passively diffuse across the enterocytic brush border of the intestine [49]. As a lipophilic molecule, it may passively diffuse across the lipid bilayer. Glycosidic cleavage occurs via β-glycosidase and lactase phlorizin hydrolase enzymes, both of which are found along intestinal epithelial cells. If it remains a glucoside, quercetin enter enterocytes via glucose transporters, including sodium dependent glucose transporter-1 and glucose transporter. Quercetin metabolism is thereby dependent on the availability, both structurally and functionally, of several enzymes including glycosidases and hydrolases, which may vary in individuals due to genotype or phenotype differences across the population. Metabolism may also be affected in people with altered gastrointestinal (GI) anatomy, including individuals who have undergone a colectomy, ileostomy, or gastric bypass, as well as those affected by GI distress, which may include altered brush border functioning as is seen in some cases of celiac disease, ulcerative colitis, or Crohn disease. 

Quercetin undergoes significant metabolism in the gastrointestinal tract, followed by hepatic metabolism. Phase I metabolism includes oxidation, reduction and hydrolysis, necessitating cytochrome P450 enzymes. Phase II metabolism follows, and includes glucuronidation, sulfation, and methylation in the liver. Given that many isoforms these enzymes may exist, particularly variations across the cytochrome P450 family of enzymes, metabolism of quercetin is highly variable across populations. Quercetin metabolites are subsequently released from liver into the bile and excreted via the large intestine.

The GI microenvironment is also likely to alter quercetin metabolism, given the synergistic effects of many biomolecules on physiological functioning. Altered by diet, genes, or other influences, an individual’s microbiome cannot be excluded from the list of variables in quercetin bioavailability.

Quercetin distribution is poorly studied. Some animal studies indicate an accumulation of quercetin primarily in the liver and kidney following a 3-day treatment of 500 mg/kg, with moderate levels found in plasma and lower levels observed in the brain, heart, and spleen. Several limitations to these studies exist, however, including limited data on humans and an uncertainty on the use of total quercetin measurements verses the measurement of aglycone alone. 

### Safety Consideration of Quercetin Treatment

Natural quercetin is reportedly well tolerated and is deemed by the FDA as a “Generally Recognized as Safe” compound. However, little is known about the long-term effects of isolated quercetin administration, particularly at high concentrations. Future studies may investigate the tolerability and potential off-target and adverse effects of quercetin treatment, as well as its ability to contribute to interaction effects. Although our study shows that quercetin may provide protection in acute TBI to cerebral endothelial cells in both cell culture and murine models, its safety profile and potential drug interaction effects must be thoroughly vetted.

### Study Limitations

The goal of our study was to investigate if quercetin provides BBB protection against ROS-induced hyperpermeability following acute TBI. Our results demonstrate that quercetin can protect BBB endothelial cells from junctional dislocalization and pial vessel hyperpermeability. One limitation to this work is our use of murine models, both in vitro and in vivo*.* Although murine models are a gold standard in basic science research, their translational applications are limited. Secondly, our studies used both rat brain endothelial cells in vitro and mice models in vivo*.* Although we believe this strengthens our findings to apply across two species, it should be noted that the models were not homogenous. Thirdly, our goal was to provide evidence for the use of quercetin in the acute time period following head injury. TBI has a widely complex pathology in which primary injury, or the initial mechanical damage to the brain parenchyma and disruption to the BBB, is followed by secondary injury, governed by several parallel and interacting pathways. Our study findings are limited to primary TBI injury and do not address the complexities of secondary injury in TBI. Fourthly, because of this acute time period, our study was designed as a terminal study, not a survival study. As such, neurocognitive and behavioral assessments were not conducted. Future work investigating TBI secondary injury should investigate the role of quercetin on functional outcomes as well as weigh the benefits and risks of various administration routes to increase delivery to the central nervous system. Although much remains to be uncovered, our study adds valuable understanding to the role of quercetin in mitigating BBB dysfunction in an acute TBI context.

## Conclusions

In conclusion, our studies have shown that oxidative stress induced by TBI results in dysfunction of the BBB via disruption of tight junctions, adherens junctions, and disorganization of the actin cytoskeleton. Administration of quercetin, a plant antioxidant compound, protected against these effects, restoring endothelial cell junctional integrity and cytoskeletal integrity and mitigating pial vessel hyperpermeability. Quercetin may be considered as a protective agent for the management of cerebral oedema and increased intracranial pressure following acute TBI. 

## Source of support

This study was funded by institutional support.
